# Health-Related Quality of Life Reported by Patients With Chagas Disease: A Systematic Review of Qualitative Evidence With GRADE Recommendations

**DOI:** 10.1590/0037-8682-0377-2022

**Published:** 2022-12-16

**Authors:** Whesley Tanor Silva, Lucas Frois Fernandes Oliveira, Diego Mendes Xavier, Pedro Henrique Scheidt Figueiredo, Ana Cristina Rodrigues Lacerda, Vanessa Amaral Mendonça, Matheus Ribeiro Ávila, Cláudio Luiz Ferreira, Márcia Maria Oliveira Lima, Mauro Felippe Felix Mediano, Manoel Otávio da Costa Rocha, Henrique Silveira Costa

**Affiliations:** 1 Universidade Federal dos Vales do Jequitinhonha e Mucuri, Programa de Pós-Graduação em Reabilitação e Desempenho Funcional, Diamantina, MG, Brasil.; 2 Universidade Federal dos Vales do Jequitinhonha e Mucuri, Departamento de Fisioterapia, Diamantina, MG, Brasil.; 3 Universidade Federal de Minas Gerais, Curso de Pós-graduação em Infectologia e Medicina Tropical, Belo Horizonte, MG, Brasil.; 4 Fundação Oswaldo Cruz, Instituto Nacional de Infectologia Evandro Chagas, Rio de Janeiro, RJ, Brasil.

**Keywords:** Chagas disease, Quality of life, Qualitative research, Systematic review

## Abstract

Patients with Chagas disease have reduced health-related quality of life (HRQoL). Hence, we aimed to identify the factors that mostly affected their HRQoL. This was a systematic review of qualitative studies. The Latin American and Caribbean Health Sciences Literature, Medical Literature Analysis and Retrieval System Online, Excerpta Medica Database, Web of Science, and SciVerse Scopus databases were searched for relevant studies without language or date restrictions. The search and data analysis were performed by independent reviewers; all qualitative studies that reported the factors that had an impact on the HRQoL of patients with Chagas disease were included. The risk of bias was assessed using the Critical Appraisal Skills Program Qualitative Study Checklist; confidence in the evidence was evaluated using the Grading of Recommendations Assessment, Development and Evaluation-Confidence in the Evidence from Reviews of Qualitative approach. Five studies were included in this review: four in Brazil and one in California, United States, with immigrants from Central and South America. The sample consisted of 207 patients with chronic Chagas disease. Stigma, physical limitations, work absenteeism, emotional or mental aspects, fear of treatment, and fear of the future had the strongest impact on the HRQoL. All items showed moderate confidence except for fear of treatment (low confidence). The physical, emotional, mental, and cultural aspects affected the HRQoL of patients with chronic Chagas disease. Identification of these factors is important in the development of strategies aimed at improving the HRQoL of this population.

## INTRODUCTION

Chagas disease (CD) was first described by the Brazilian scientist Carlos Chagas in 1909^1^ ^,^ ^2^. It remains a public health problem in Latin America^3^, with a potential expansion to non-endemic countries^4^. The acute phase is characterized by non-specific clinical manifestations^5^. Patients later progress to the chronic phase, which can present in indeterminate form (no specific signs and symptoms of disease)^6^, cardiac form (with heart disease), digestive form (with digestive impairment), or mixed form (simultaneous cardiac and digestive involvement)^7^.

Subjective health assessments have gained importance for clinicians, health policymakers, and researchers^8^, especially health-related quality of life (HRQoL). In the setting of CD, many studies have quantitatively evaluated the HRQoL of patients^9^ ^-^ ^13^. A recent state-of-the-art study^14^ summarized the current findings and postulated that HRQoL is worse in patients with CD compared with that in healthy individuals. Moreover, HRQoL is more compromised in women with cardiovascular and gastrointestinal symptoms among patients with worse functional class, lower levels of physical activity, and worse cardiac function. Other studies have shown that HRQoL has potential value in screening patients with left systolic dysfunction^15^ and identifying those at an increased risk of developing adverse cardiovascular events^13^, which highlights the value of HRQoL in the clinical follow-up of patients. 

Despite evidence from previous studies, quantitative data are based on the participants’ response to the questionnaire-based survey with predefined questions, limiting the patient’s freedom of response. The major factors that affect the HRQoL of patients with CD should be determined. The identification of these factors can be carried out through qualitative interview^16^ and can guide both clinicians and researchers in the selection of outcomes of patients’ management^17^ ^-^ ^19^. However, to the best of our knowledge, no qualitative studies have evaluated on the CD patients’ perception of the items that impact their HRQoL. Hence, the present study aimed to systematically review qualitative studies that investigated the items that has an impact on the HRQoL of patients with CD.

## METHODS

### Study design

This systematic review of qualitative studies aimed to identify the factors reported by CD patients that mostly affected their HRQoL and are part of the Caburé project (collaborative project focusing on social, historical, and geographical aspects of CD, which aims to create a specific instrument to assess patients’ HRQoL). The study was previously registered in the Open Science Framework (https://osf.io/4mk9u/) and edited following the Enhancing Transparency in Reporting the Synthesis of Qualitative Research Guidelines, an instrument used to describe the synthesis of qualitative research in health sciences^20^.

### Peer review method

As recommended by Cochrane^21^, the screening of potentially eligible studies, data extraction, risk of bias assessment, and assessment of the quality of current evidence were performed by two independent reviewers: WTS and LFFO performed the screening of potentially eligible studies, data extraction, and risk of bias assessment, while WTS and DMX used the Grading of Recommendations Assessment, Development and Evaluation (GRADE) to assess the quality of current evidence. Discrepancies were resolved by consulting a third reviewer (HSC).

### Searches and study selection

Potentially eligible studies were identified using a systematic search. The databases used were Latin American and Caribbean Health Sciences Literature (LILACS), Medical Literature Analysis and Retrieval System Online (MEDLINE), Excerpta Medica Database, Web of Science, and SciVerse Scopus for relevant studies. The search terms used were related to “Chagas disease,” “quality of life,” and “qualitative studies.” Each database was searched from the date of inception until November 2021 and updated in July 2022.

We manually reviewed the full-text versions of relevant systematic reviews and used a manual search to retrieve records from gray literature. The strategy used to search the MEDLINE database was [(“American Trypanosomiasis” OR “Trypanosomiasis, American” OR “Trypanosomiasis, South American” OR “South American Trypanosomiasis” OR “Trypanosoma cruzi Infection” OR “Infection, Trypanosoma cruzi” OR “Infections, Trypanosoma cruzi” OR “Trypanosoma cruzi Infections” OR “Chagas' Disease” OR “Cardiomyopathy, Chagas” OR “Trypanosomiasis, Cardiovascular” OR “Cardiovascular Trypanosomiasis” OR “Chagas Cardiomyopathy” OR “Cardiomyopathy, Chagas'” OR “Myocarditis, Chagas”) AND (“Quality of Life” OR “Life Quality” OR “Health-Related Quality Of Life” OR “Health-related Quality Of Life” OR “HRQOL”)]. More details about the search strategy are provided in the Supplementary Material. After the search, the retrieved references were exported to Endnote®, and duplicate studies were removed. The authors screened the titles and abstracts and evaluated the full texts. Studies that met the eligibility criteria were included in this review. 

### Inclusion criteria

Primary qualitative studies that identified the factors that impact the HRQoL of individuals of both sexes, regardless of age, with at least two positive serological tests for *Trypanosoma cruzi* performed in any healthcare setting were included in this review. There were no restrictions on the language or year of publication.

### Data extraction

Data on (a) the characteristics of the sample, (b) process of data collection and sources, (c) results of data analysis, and (d) main findings related to the HRQoL of patients were extracted. When data from a study were unavailable or unpublished, the original investigators were contacted to request the information. When necessary, the authors were contacted three times, with an interval of one week.

### Risk of bias assessment

All included studies were assessed for risk of bias by reviewers using the Critical Appraisal Skills Program (CASP) Qualitative Study Checklist^22^. The CASP checklist composed of 10 questions, and the response options included “yes,” “no,” or “can’t tell”^23^.

### Data analysis

The meta-synthesis method developed by Noblit and Hare was performed according to the instructions described by Britten et al.^24^. We present a meta-theory that demonstrates the analysis and interpretation of different theoretical and philosophical perspectives, sources, and assumptions shown in individual studies. Therefore, resynthesis was performed based on the meta-synthesis of the findings. 

The characteristics and relationships between them were defined (location, sample characteristics, type of collection, and stage of disease). With information compiled based on the results of previous studies and researchers’ experience, the most frequent findings were grouped. Finally, the narrative summary technique was used, presenting the re-synthesis of the information collected and justifying the items that impact the HRQoL of patients with CD, as recommended by the Cochrane Handbook^25^.

The quality of current evidence was verified using the GRADE-Confidence in the Evidence from Reviews of Qualitative Research (GRADE-CERQual) approach^26^. According to this system, the evidence is classified as having high, moderate, low, or very low confidence. High-confidence evidence is related to a finding likely to be a reasonable representation of the phenomenon of interest, while low-confidence evidence shows that it remains unclear whether the finding is a representation of the phenomenon of interest, thus requiring further study^26^. More details regarding the confidence assessment are provided in the Supplementary Material.

## RESULTS

**FIGURE 1:**
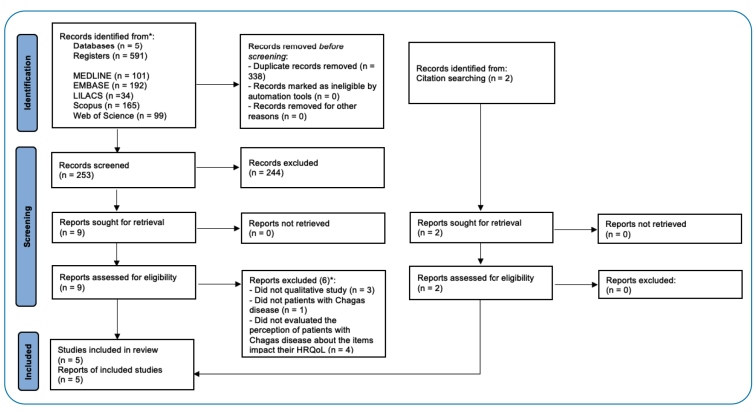
Flowchart showing the process of selecting the studies for review. *Articles could be excluded for more than one reason.

An electronic search strategy identified 591 relevant studies based on their titles and abstracts. Of these, 338 (57.2%) were duplicates. The remaining 253 studies were screened, and 9 references were selected as potentially eligible studies, of which 6 were excluded. After a manual search of previous reviews, we identified two other articles for full-text assessment, and both were included. Finally, five studies met the inclusion criteria^27^ ^-^ ^31^. Figure 1 outlines the process of selecting relevant articles for review. 

The included studies were published between 2000 and 2021. Four were carried out in Brazil and one in California, United States, with immigrants from Central and South America. Among the included studies, the sample size ranged from 1 to 131 patients, and the total sample consisted of 207 individuals, all in the chronic phase of the disease. The ages of the patients ranged from 27 to 82 years. 

The characteristics of the studies and their relationships are presented in Table 1. The meta-theory, with the main issues addressed by individual studies, is presented in Table 2. The details of the resynthesis (meta-synthesis) are described in the text. In summary, the issues that could impact the HRQoL of patients were grouped into 1) stigma, 2) mental health and/or emotional aspects, 3) physical limitations and work absenteeism, 4) fear of the future, and 5) fear of treatment. Results of the risk of bias assessment are shown in Table 3. Results of the assessment of the confidence of the findings for each thematic group performed using the GRADE-CERQual approach are shown in Table 4.

**TABLE 1:** Baseline characteristics of all included studies and the relationship between them (n=5).

Study (year)	Country	Sample characteristics	Data collection	Disease stage
Araújo *et al.,* 2000^27^	Brazil	n=131, aged from 31 years to 60 years, 62 men and 69 women	Individual interview	Chronic phase
Magnani, Oliveira, Gontijo, 2007^28^	Brazil	n=15, aged from 27 years to 75 years, 8 men and 7 women	Individual interview	Chronic phase
Ballester-Gil *et al.*, 2008^29^	Brazil	n=1, aged 66 years, woman	Semi-structured individual interview	Chronic phase
Oliveira *et al.*, 2010^30^	Brazil	n=10, aged from 51 years to 82 years, sex not reported	Semi-structured individual interview	Chronic phase
Forsyth *et al.,* 2021^31^	United States	n=50, 17 men and 33 women	Semi-structured individual interview	Chronic phase

**TABLE 2:** Interpretation of the different theoretical and philosophical perspectives (n=5).

Study (year)	Theoretical and philosophical perspectives
Araújo *et al.,* 2000^27^	It presents reports about the history of life and the disease, such as the existence of other cases in the family, what they felt when receiving the diagnosis, and impact on life after knowing the diagnosis.
Magnani, Oliveira, Gontijo, 2007^28^	It presents a theory about the symbology of Chagas disease in the lives of patients who are infected.
Ballester-Gil *et al.*, 2008^29^	It presents the patient’s perceptions and knowledge about Chagas disease, which refer to the form of diagnosis; the feeling at the time of diagnosis; perceptions about the disease; perceptions about life in recent years; and perceptions about the forms of consultation and treatment.
Oliveira *et al.*, 2010^30^	It presents information based on the patient’s perspective of the disease vector and on how they live and how they face the symptoms of the pathology.
Forsyth *et al.,* 2021^31^	It presents the theory about patients’ knowledge of CD, impact of diagnosis (e.g., emotional), symptoms, access to treatment, strategies for treating the disease, sociopolitical context of CD, and adaptation to life in the United States, thus concluding the disease as neglected.

**TABLE 3:** Risk of study bias.

	Risk of bias									
Study (year)	1) Was there a clear statement of the aims of the research?	2) Is a qualitative methodology appropriate?	3) Was the research design appropriate to address the aims of the research?	4) Was the recruitment strategy appropriate to the aims of the research?	5) Was the data collected in a way that addressed the research issue?	6) Has the relationship between researcher and participants been adequately considered?	7) Have ethical issues been taken into consideration?	8) Was the data analysis sufficiently rigorous?	9) Is there a clear statement of findings?	10) How valuable is the research?
Araújo *et al.,* 2000^27^	yes	Can’t tell	no	yes	no	no	yes	no	no	yes
Magnani, Oliveira, Gontijo, 2007^28^	yes	yes	yes	no	yes	no	yes	yes	no	yes
Ballester-Gil *et al.*, 2008^29^	yes	yes	yes	no	yes	yes	yes	yes	no	yes
Oliveira *et al.*, 2010^30^	yes	yes	yes	yes	yes	no	yes	yes	no	yes
Forsyth *et al.,* 2021^31^	yes	yes	yes	yes	yes	yes	yes	yes	no	yes

**TABLE 4:** Confidence in the evidence according to the GRADE-CERQual scores.

Items that impact the HRQoL of patients with Chagas disease	CERQual Assessment of Confidence in the Evidence	Explanation of CERQual Assessment	Studies Contributing to the Review Finding
Stigma impacts the HRQoL of patients with CD.	Moderate confidence	Minor concerns in the methodological quality, coherence, data adequacy, and relevance	de Araújo *et al*., 2000
			Magnani *et al.*, 2007
Mental/emotional component impacts the HRQoL of patients with CD.	Moderate confidence	Minor concerns in the methodological quality and coherence; major concerns in the data adequacy and relevance	de Araújo *et al*., 2000
			Ballester-Gil *et al.*, 2008
			Forsyth *et al.*, 2021
The physical component and work absenteeism impact the HRQoL of patients with CD.	Moderate confidence	Minor concerns in the methodological quality, coherence, data adequacy, and relevance	Ballester-Gil *et al.*, 2008
			De Oliveira *et al.*, 2010
			Forsyth *et al.*, 2021
Fear of the future impacts the HRQoL of patients with CD.	Moderate confidence	Minor concerns in the methodological quality, coherence, and relevance. Major concerns in the data adequacy	de Araújo *et al.*, 2000
			Ballester-Gil *et al.*, 2008
			Magnani *et al.*, 2007
			Forsyth *et al.*, 2021
Fear of treatment impacts HRQoL of patients with CD.	Low confidence	Minor concerns in the methodological quality and relevance; major concerns in the data adequacy and coherence	Ballester-Gil *et al.*, 2000
			Forsyth *et al.*, 2021

Abbreviations: **CD:** Chagas Disease; **CERQaul:** Confidence in the Evidence from Reviews of Qualitative Research; **HRQoL:** Health-related quality of life.

### Impact of stigma on HRQoL

Stigma is a situation in which the individual is aware of what is attributed to him by society and then begins to agree and apply negative stereotypes about the illness to oneself^32^. In the present review, two studies identified the impact of stigma on HRQoL^27^ ^,^ ^28^ (moderate confidence).

In one study^27^, patients reported fear and shame, leading them to hide the disease from others: “Nobody knows at my job” and “Look, I am here now, but no one in my family knows I have *this* disease.” Another study^28^ also described the interview response of an individual related to the stigma of sudden cardiac death: “People say these things: That Chagas disease... So-and-so died of Chagas disease, who would fall suddenly and die. This is how we heard about it. However, you know that even today, I still have it in my head from time to time. (...) This for me is death alone. Even that I think, do you understand? They say that whoever has Chagas disease, the person who is like this, falls and dies suddenly!”

### Impact of mental health and/or emotional aspects on HRQoL

Three studies included interviews related to the mental health and/or emotional aspects of HRQoL (moderate confidence)^27^ ^,^ ^29^ ^,^ ^31^. One of the studies^27^ demonstrated the emotional burden of having the disease: “Having Chagas disease for me is a problem.” In addition, other responses showed that the mental and/or emotional impact was detected since the diagnosis: “I love to dance, but after I found out I had Chagas disease I thought I had to go home, lie down, and wait for death...,” and “I felt very sad, lost, when I found out I had a disease that has no cure.” 

Another study^29^ reported the impact of CD on the mental and/or emotional aspects of HRQoL: “I feel very sad. I cry a lot. I am feeling down. I do not feel like talking or seeing anyone. I keep thinking that my heart is swollen and I feel more things than before. My life changed.”, and “I'm shaken, I don't want to talk to anyone. I am alone, thinking that I might die. I cry a lot every day, and I get out of control. I cry until I run out of air. I do not know how I had the strength to come today.” Finally, “this difficulty and pain caused me much sadness. I feel my heartbeat is fast.” 

In a previous study^31^, the authors also verified the impact of mental and/or emotional factors on HRQoL: “I became depressed, I felt very sad, because you don’t know if you’re going to die tomorrow or what … I felt down, sad, without a desire to do anything….”

### Impact of the physical component and work absenteeism on HRQoL

In several cases, patients with CD are removed from their jobs, particularly those with the cardiac form of the disease^33^ ^,^ ^34^. Physical limitations are one of the components that contribute to restriction in daily activities^35^, as observed in three studies^29^ ^-^ ^31^ (moderate confidence).

In one of them, Ballester-Gil *et al*.^29^ highlighted the impact of some physical factors related to body structure and function on patients’ HRQoL: “My heart is different. The patient’s blood pressure was high. I have trouble swallowing, and I have pain near my stomach. It feels like a hernia. I feel weak; I feel weak. When I eat, I feel weaker. When I eat. I have a hernia in my esophagus. An umbilical hernia. I have varicose ulcer. I have osteoporosis and arthrosis.”

Corroborating these findings, Forsyth *et al.* ^31^ interviewed individuals who reported physical complaints such as shortness of breath, weakness, and tiredness. The patients also reported that the presence of physical symptoms compromised their HRQoL: “There were strong palpitations that did not stop, and I felt my heart was very large. It is very uncomfortable because you do not have time to sleep or anything; you cannot relax with the constant jumping of your heart that is not at all normal.”

Another study^20^ showed the impact of this disease on work activities and, consequently, on the patient’s HRQoL. The physical limitations are highlighted in the following statements: “[...] I cannot do anything I did before because everything tires me a lot and makes my chest feel tight and suffocate! If I didn’t have the disease, I would be working, I had to stop because of that”, “[...] Today I can’t work in the same way as before, it's exhausting, I feel weak and I have to rest.” and “Yes at home For hours, I cannot do things because of the pain; I feel unwell.” 

### Impact of fear of the future on HRQoL

Among the possible adverse events in patients with CD, sudden cardiac death may be the initial and/or most important manifestation of the disease^36^. Sudden cardiac death is the result of electrophysiological disturbances due to myocardial damage caused by the disease^37^. When people close to them who had CD suddenly died, patients with the disease experience extreme fear and insecurity about the future, especially of dying at any time, as reported in three studies^28^ ^,^ ^29^ ^,^ ^31^ (moderate confidence).

One of the aforementioned studies^28^ demonstrated reports about fear of the future: “Sometimes I was standing up like this and falling backward. This is Chagas disease. All the patients died suddenly. I would lie down to sleep, talk, and never get up,” or “I always heard: Chagas, Chagas (...) They say that whoever has Chagas, the person who is like that suddenly falls and dies! And it’s horrible! It’s like saying: ‘You have cancer!’ (...) It was like that for me at the time. Because I said: Gee, I’m doomed, I’m going to die soon,” and “People say these things: That Chagas... So-and-so died of Chagas, who would fall suddenly and die. This is how we heard about it. However, you know that even today, I still have it in my head from time to time. (...) This for me is death alone. Even that I think, do you understand? They say that whoever has Chagas, the person who is like this, falls down and dies suddenly!”

Ballester-Gil *et al.* ^29^ also reported some findings that corroborate the presence of fear of the future experienced by patients with CD: “I know this disease has no cure. I know I will die. I am sad; my life has changed since I got the result. I heard that Chagas disease has no cure, and I know I am going to die. The doctor explained how the disease and the exams were, but even though he was really polite and comforted me a lot, I know I am going to die.” Forsyth *et al*.^31^ also observed interviews wherein patients with CD showed fear of the future and death: “I don’t want to die, I’m only 57. I want to see my family and my grandchildren grow.” and “Dying is what worries me. It has been 15 years since I am sick. I do not get better, but I do not die. But that’s my fear; I am getting on in age and I am always sick.”

### Impact of Fear of treatment on HRQoL

CD tends to progress, and the available medications can be toxic and cause adverse side effects. Additionally, patients require permanent therapeutic measures, some of which are invasive, such as cardiac pacemakers. Two studies^29^ ^,^ ^31^ highlighted the impact of fear of treatment on patients’ HRQoL (low confidence).

In a previous study^29^, the patients reported, “I’m all complicated. Where I am most nervous is because of this. I am afraid to put on a pacemaker. We see and hear on the television. I know that there is no cure. The pacemaker is a surgery to improve, but it has harmful effects. A pacemaker case can be a great success for a person, but it can also have serious consequences. Then, I get nervous about it. Besides, I have so many [problems] that if I did not have any of these businesses or other problems. If I were a healthy woman, and that's all, I would not have bothered to put a marker on. I am allergic, so I am afraid of everything and of putting a marker on that. And not feeling better and making my life worse.” And “Well, I asked the doctor and he said that tests are necessary, depending on the case and, in my case, depending on the state, I will have to put pacemaker. I know about the effects of the pacemaker on the person; it is very bad and the surgery is complicated.” 

In addition to the fear of undergoing cardiac pacemaker implantation, drug treatment also caused fear in patients, as demonstrated in statements in the study of Forsyth *et al.* ^31^: “From so much medicine you get scared, it frightens you, but you don’t want to die either, right? And sometimes I feel panicked, I feel scared, but the truth is I did the treatment because I do not want to die, I want to live, and thanks to it I have lived longer.”

## DISCUSSION

This qualitative review is the first to identify the factors that impact the HRQoL of patients with CD, demonstrating that stigma, emotional and/or mental components, physical aspects and absence from work, fear of the future, and fear of treatment mostly affect the HRQoL of patients with chronic CD.

CD is among the neglected tropical diseases^38^. This phenomenon is caused by complex social, interpretive, and cultural processes^39^. Stigma is considered harmful to health and can cause intense psychosocial problems^40^, thus becoming a major concern of healthcare authorities and epidemiologists^41^. The stigma may be due to several sociocultural reasons that remain uncertain, but it may be related to work, where patients feel the fear of being terminated for having the disease or even insecurity of not being approved during the hiring interview. Studies on other health conditions have shown stigma as a marker of worse prognosis^42^ ^-^ ^44^, and people with CD have a greater tendency to be submissive and attacked by other people^45^. Therefore, due to the impact of stigma on the HRQoL of patients with CD, the present review reinforces the need to bring patients into the community, especially within the scope of primary healthcare. In addition to expanding public policies, encouraging conversations and health education strategies aimed at this neglected population should be implemented, particularly in endemic areas. 

In addition to stigma, physical complaints and limitations at work can also affect the HRQoL of these patients. Physical limitations are noticeable in patients with the cardiac form of the disease, mainly due to progressive fatigue and dyspnea^46^. A systematic review and meta-analysis^47^ showed that a reduction in exercise capacity is detectable from the early stages of heart disease; hence, it is necessary to stimulate strategies to improve the physical status of this population. Moreover, physical limitations are associated with worse levels of HRQoL^48^ and are closely related to restrictions on activities and absence from work^34^. Workers with CD experience several adverse situations, such as a high rejection rate among job applicants and non-admission after showing positive results on pre-physical examinations or serological tests for the disease, regardless of the clinical status, in addition to being subject to dismissal without just cause^49^. Leaving from work predisposes patients with CD to future limitations as well as disability and marginalization, being part of a cycle between removal and increased inability to work^50^. International declarations consider work as a right for all and must be chosen by the individual under fair and favorable conditions^51^. However, this right has not been granted to the patients with CD^49^. In some cases, absence from work may occur as a result of physical impairments, which can affect the worker’s productivity and ability to perform work safely^35^.

Moreover, fear of the future can impact patients’ HRQoL. This can be explained by the prognosis of the condition, which may progress to megaesophagus, megacolon, esophageal motor disorders, diarrhea, and abdominal distension^52^, cardiac dysfunction^53^, functional impairment^47^, sudden cardiac death^36^, stroke, and heart transplantation^54^. With regard to fear of treatment, some patients reported fear of undergoing a device implantation such as a cardiac pacemaker. The implantation of a cardiac pacemaker may indicate having a weak heart or irregular beats^28^. Drug treatment was also pointed out as a reason for fear among this population, which is probably due to the possibility of severe adverse side effects caused by use of trypanocidal drugs (benznidazole and nifurtimox), such as cutaneous rash, gastrointestinal symptoms, and peripheral polyneuropathy^55^ ^-^ ^57^. In this scenario, communication is required between healthcare professionals and patients, especially regarding health education strategies. With empowerment and awareness of the prognosis and effects of treatment, patients can face the disease with a better HRQoL.

Finally, mental and/or emotional factors are important aspects of the HRQoL of patients with CD, as demonstrated by the high rate of depressive symptoms in the population^11^ ^,^ ^58^. The psyche is constructed through a complex interaction between the biological apparatus and social and cultural factors^59^. Stigma, fear of death, and inability to perform the activities of daily living are personal and environmental factors that can contribute to these symptoms^60^. Thus, the management of patients with chronic CD should not only involve the clinical but also the psychological aspects.

The present study has several strengths, limitations, and perspectives. In terms of strengths, the items that impact the HRQoL of patients with CD were systematically identified for the first time and the review was performed according to the suggested methodology. The results are of great clinical and scientific significance, as they can guide both the goals of clinical management and researchers’ choice of outcomes when performing new interventions in patients with CD.

As a limitation, all included studies selected patients in the chronic phase of the disease without stratifying the clinical form. Grouping patients with the indeterminate form together with patients with cardiac and digestive forms can be inappropriate because the needs, signs and symptoms, limitations, and prognoses can be different, which affects the HRQoL. Almost all studies were conducted in Brazil. Hence, further studies should be conducted in other Latin American countries, where the condition is highly prevalent and socioeconomic and cultural conditions are very heterogeneous^60^. Finally, one potentially eligible study was not evaluated owing to the lack of data for analysis. The author was contacted, but did not respond to the main researcher of the present review.

Therefore, a specific HRQoL assessment questionnaire for CD should be developed. Many of the factors identified in the present review that impact HRQoL, such as stigma and fear of treatment, were not addressed by the survey using generic questionnaires. Researchers have used generic questionnaires, such as the Short-Form Health Survey (SF-36), Abbreviated World Health Organization Quality of Life (WHOQoL-BREF), Minnesota Living with Heart Failure Questionnaire, Assessment of Quality of Life, and RELated Events^48^. Despite their wide use, they are not commonly used in assessing patients with CD. Therefore, the development of a specific questionnaire for assessing this disease is required to improve the assessment and monitoring of HRQoL in patients with CD.

Stigma, physical limitations, absence of work, mental and/or emotional aspects, fear of the future, and fear of treatment are factors cited by qualitative studies that most impact the HRQoL of patients with CD. Results showed moderate confidence and, despite these limitations, can help in clinical and scientific decision-making.
