# Supplementary material for: Health-Related Quality of Life Reported by Patients With Chagas Disease: A Systematic Review of Qualitative Evidence With GRADE Recommendations
Source: Rev Soc Bras Med Trop. 2022 Dec 16;55:e0377-2022. doi: 10.1590/0037-8682-0377-2022 (PMC9757717; doi:10.1590/0037-8682-0377-2022)
Supplement: Supplementary file 1 [file 1678-9849-rsbmt-55-e0377-2022-supp1.pdf]

Search Strategy and GRADE assessment

Health-related quality of life reported by patients with Chagas disease: a systematic review of qualitative evidence with GRADE recommendations

Search strategy

Search	Query	Records retrieved
**MEDLINE (Pubmed)**	[("American Trypanosomiasis" OR "Trypanosomiasis, American" OR "Trypanosomiasis, South American" OR "South American Trypanosomiasis" OR "Trypanosoma cruzi Infection" OR "Infection, Trypanosoma cruzi" OR “Infections, Trypanosoma cruzi” OR “Trypanosoma cruzi Infections” OR “Chagas' Disease” OR “Cardiomyopathy, Chagas” OR “Trypanosomiasis, Cardiovascular” OR “Cardiovascular Trypanosomiasis” OR “Chagas' Cardiomyopathy” OR “Cardiomyopathy, Chagas'” OR “Myocarditis, Chagas”) AND ("Quality of Life" OR "Life Quality" OR "Health-Related Quality Of Life" OR "Health Related Quality Of Life" OR "HRQOL")]	101
**EMBASE**	# 1 'chagas disease'/exp OR 'american trypanosomiasis' OR 'chagas disease' OR 'chagas infection' OR 'chagas mazza disease' OR 'chagas`s disease' OR 'trypanosoma cruzi infection' OR 'disease, chagas' OR 'infection by trypanosoma cruzi' OR 'infection of trypanosoma cruzi' OR 'south american trypanosomiasis' OR 'trypanosomiasis, american' OR 'trypanosomiasis, south american' OR 'chagas cardiomyopathy'/mj OR 'chagas cardiomyopathy' OR 'chagas disease cardiomyopathy' OR 'chagas heart disease' OR 'chagas myocardiopathy' OR 'chagas myocarditis' OR 'cardiomyopathy in chagas disease' OR 'chronic chagas disease cardiomyopathy'<br># 2 'quality of life'/mj OR 'hrql' OR 'health related quality of life' OR 'life quality' OR 'quality of life' OR 'quality of life assessment'/mj<br>#1 AND #2	192
**LILACS**	"Doença de Chagas " OR "Chagas Disease" OR "Enfermedad de Chagas" OR "Maladie de Chagas" AND "Qualidade de Vida" OR "Quality of Life" OR "Calidad de Vida" OR "Qualité de vie" OR "HRQOL" OR "QVRS" OR "Qualidade de Vida Relacionada à Saúde"	34
**Scopus**	(TITLE-ABS-KEY (("American Trypanosomiasis" OR "Trypanosomiasis, American" OR "Trypanosomiasis, South American" OR "South American Trypanosomiasis" OR "Trypanosoma cruzi Infection" OR "Infection, Trypanosoma cruzi" OR "Infections, Trypanosoma cruzi" OR "Trypanosoma cruzi Infections" OR "Chagas' Disease" OR "Cardiomyopathy, Chagas" OR "Trypanosomiasis, Cardiovascular" OR "Cardiovascular Trypanosomiasis" OR "Chagas' Cardiomyopathy" OR "Cardiomyopathy, Chagas'" OR "Myocarditis, Chagas")) AND TITLE-ABS-KEY (("Quality of Life" OR "Life Quality" OR "Health-Related Quality Of Life" OR "Health Related Quality Of Life" OR "HRQOL")))	165
**Web of Science**	(ALL=("American Trypanosomiasis" OR "Trypanosomiasis, American" OR "Trypanosomiasis, South American" OR "South American Trypanosomiasis" OR "Trypanosoma cruzi Infection" OR "Infection, Trypanosoma cruzi" OR “Infections, Trypanosoma cruzi” OR “Trypanosoma cruzi Infections” OR “Chagas' Disease” OR “Cardiomyopathy, Chagas” OR “Trypanosomiasis, Cardiovascular” OR “Cardiovascular Trypanosomiasis” OR “Chagas' Cardiomyopathy” OR “Cardiomyopathy, Chagas'” OR “Myocarditis, Chagas”)) AND ALL=("Quality of Life" OR "Life Quality" OR "Health-Related Quality Of Life" OR "Health Related Quality Of Life" OR "HRQOL")	98
**Total**		590

Search strategy conducted in the databases with limits and filters, when used. Literature updates were monitored by email alerts. Search conducted on November 24, 2021. Strategy reported according to PRISMA-S: an extension to the PRISMA Statement for Reporting Literature Searches in Systematic Reviews

GRADE-CERQual assessment of confidence in the results of the evidence synthesis

Evaluation of the components of the CERQual approach

Stigma impacts the HRQoL of patients with CD

Methodological limitations

	NO WORRIES OR VERY MINOR	MINOR WORRIES	MODERATE CONCERNS	SERIOUS WORRIES
ARAÚJO et al. 2000			X	
MAGNANI et al. 2007		X		

Coherence

	NO WORRIES OR VERY MINOR	MINOR WORRIES	MODERATE CONCERNS	SERIOUS WORRIES
ARAÚJO et al. 2000			X	
MAGNANI et al. 2007	X			

Data adequacy

	NO WORRIES OR VERY MINOR	MINOR WORRIES	MODERATE CONCERNS	SERIOUS WORRIES
ARAÚJO et al. 2000			X	
MAGNANI et al. 2007				x

Relevance

	NO WORRIES OR VERY MINOR	MINOR WORRIES	MODERATE CONCERNS	SERIOUS WORRIES
ARAÚJO et al. 2000			X	
MAGNANI et al. 2007			X	

Mental/emotional component impacts the HRQoL of patients with CD.

Methodological limitations

	NO WORRIES OR VERY MINOR	MINOR WORRIES	MODERATE CONCERNS	SERIOUS WORRIES
BALLESTER et al. 2008		X		
FORSYTH, COLIN J. et al. (2021)	X			

Coherence

	NO WORRIES OR VERY MINOR	MINOR WORRIES	MODERATE CONCERNS	SERIOUS WORRIES
BALLESTER et al. 2008		X		
FORSYTH, COLIN J. et al. (2021)		X		

Data adequacy

	NO WORRIES OR VERY MINOR	MINOR WORRIES	MODERATE CONCERNS	SERIOUS WORRIES
BALLESTER et al. 2008				X
FORSYTH, COLIN J. et al. (2021)		X		

Relevance

	NO WORRIES OR VERY MINOR	MINOR WORRIES	MODERATE CONCERNS	SERIOUS WORRIES
BALLESTER et al. 2008			X	
FORSYTH, COLIN J. et al. (2021) (2021)	X			

The physical component and work absenteeism impact the HRQoL of patients with CD

Methodological limitations

	NO WORRIES OR VERY MINOR	MINOR WORRIES	MODERATE CONCERNS	SERIOUS WORRIES
BALLESTER et al., 2008		X		
OLIVEIRA et al. (2010)		X		
FORSYTH, COLIN J. et al. (2021)	X			

Coherence

	NO WORRIES OR VERY MINOR	MINOR WORRIES	MODERATE CONCERNS	SERIOUS WORRIES
BALLESTER et al. 2008		X		
OLIVEIRA et al. 2010		X		
FORSYTH, COLIN J. et al. (2021)		X		

Data adequacy

	NO WORRIES OR VERY MINOR	MINOR WORRIES	MODERATE CONCERNS	SERIOUS WORRIES
BALLESTER et al., 2008			X	
OLIVEIRA et al. 2010		X		
FORSYTH, COLIN J. et al. (2021)		X		

Relevance

	NO WORRIES OR VERY MINOR	MINOR WORRIES	MODERATE CONCERNS	SERIOUS WORRIES
BALLESTER et al. 2008;		X		
OLIVEIRA et al. 2010		X		
FORSYTH, COLIN J. et al. (2021)		X		

Fear of the future impacts the HRQoL of patients with CD

Methodological limitations

	NO WORRIES OR VERY MINOR	MINOR WORRIES	MODERATE CONCERNS	SERIOUS WORRIES
BALLESTER et al. 2008;		X		
MAGNANI et al. 2007		X		
FORSYTH, COLIN J. et al. (2021)	X			

Coherence

	NO WORRIES OR VERY MINOR	MINOR WORRIES	MODERATE CONCERNS	SERIOUS WORRIES
BALLESTER et al., 2008;	X			
MAGNANI et al. 2007		X		
FORSYTH, COLIN J. et al. (2021)		X		

Data adequacy

	NO WORRIES OR VERY MINOR	MINOR WORRIES	MODERATE CONCERNS	SERIOUS WORRIES
BALLESTER ET AL. 2008				X
MAGNANI; ET AL. 2007			X	
FORSYTH, COLIN J. et al. (2021)		X		

Relevance

	NO WORRIES OR VERY MINOR	MINOR WORRIES	MODERATE CONCERNS	SERIOUS WORRIES
BALLESTER et al., 2008		X		
MAGNANI ET AL. 2007		X		
FORSYTH, COLIN J. et al. (2021)		X		

Fear of treatment impacts HRQoL of patients with CD

Methodological limitations

	NO WORRIES OR VERY MINOR	MINOR WORRIES	MODERATE CONCERNS	SERIOUS WORRIES
BALLESTER et al., 2008;		X		
FORSYTH, COLIN J. et al. (2021)	X			

Coherence

	NO WORRIES OR VERY MINOR	MINOR WORRIES	MODERATE CONCERNS	SERIOUS WORRIES
BALLESTER et al., 2008;			X	
FORSYTH, COLIN J. et al. (2021)		X		

Data adequacy

	NO WORRIES OR VERY MINOR	MINOR WORRIES	MODERATE CONCERNS	SERIOUS WORRIES
BALLESTER et al., 2008;				X
FORSYTH, COLIN J. et al. (2021)		X		

Relevance

	NO WORRIES OR VERY MINOR	MINOR WORRIES	MODERATE CONCERNS	SERIOUS WORRIES
BALLESTER et al., 2008;		X		
FORSYTH, COLIN J. et al. (2021)		X		
